# Econometric evaluation of implementing a behavioral health integration intervention in primary care settings

**DOI:** 10.1093/tbm/ibad013

**Published:** 2023-03-31

**Authors:** Zsolt Nagykaldi, Benjamin Littenberg, Levi Bonnell, Ryan Breshears, Jessica Clifton, Abigail Crocker, Juvena Hitt, Rodger Kessler, Brenda Mollis, Robin E S Miyamoto, Constance van Eeghen

**Affiliations:** Department of Family and Preventive Medicine, University of Oklahoma Health Sciences Center, Oklahoma City, OK, USA; Department of Medicine, University of Vermont, Burlington, VT, USA; Department of Medicine, University of Vermont, Burlington, VT, USA; Psychological Services, Wellstar Health System, Marietta, GA, USA; University of Colorado School of Medicine, USA; Department of Mathematics and Statistics, University of Vermont, Burlington, VT, USA; Department of Medicine, University of Vermont, Burlington, VT, USA; Integrated Behavioral Health, Arizona State University, Phoenix, AZ, USA; Department of Family Medicine, University of Washington, Seattle, WA, USA; Departments of Native Hawaiian Health and Family Medicine and Community Health, University of Hawai’i, Honolulu, HI, USA; Department of Medicine, University of Vermont, Burlington, VT, USA

**Keywords:** Behavioral health integration, Primary care, Cost analysis, Program implementation

## Abstract

Integrated behavioral health (IBH) is an approach to patient care that brings medical and behavioral health providers (BHPs) together to address both behavioral and medical needs within primary care settings. A large, pragmatic, national study aimed to test the effectiveness and measure the implementation costs of an intervention to improve IBH integration within primary care practices (IBH-PC). Assess the time and cost to practices of implementing a comprehensive practice-level intervention designed from the perspective of clinic owners to move behavioral service integration from co-location toward full integration as part of the IBH-PC study. IBH-PC program implementation costs were estimated in a representative sample of 8 practices using standard micro-econometric evaluation of activities outlined in the implementation workbook, including program implementation tasks, remote quality improvement coaching services, educational curricula, and learning community activities, over a 24-month period. The total median cost of implementing the IBH-PC program across all stages was $20,726 (range: $12,381 - $60,427). The median cost of the Planning Stage was $10,258 (range: $4,625 - $14,840), while the median cost of the Implementation Stage was $9,208 (range: $6,017 – 49,993). There were no statistically significant differences in practice or patient characteristics between the 8 selected practices and the larger IBH-PC practice sample (N=34). This study aimed to quantify the relative costs associated with integrating behavioral health into primary care. Although the cost assessment approach did not include all costs (fixed, variable, operational, and opportunity costs), the study aimed to develop a replicable and pragmatic measurement process with flexibility to adapt to emerging developments in each practice environment, providing a reasonable ballpark estimate of costs associated with implementation to help guide future executive decisions.

## Teaser

Integration of behavioral health services into primary care does not need to cost too much—we estimated that this cost is around $20,000 per office.

## Graphical Abstract

**Figure:**
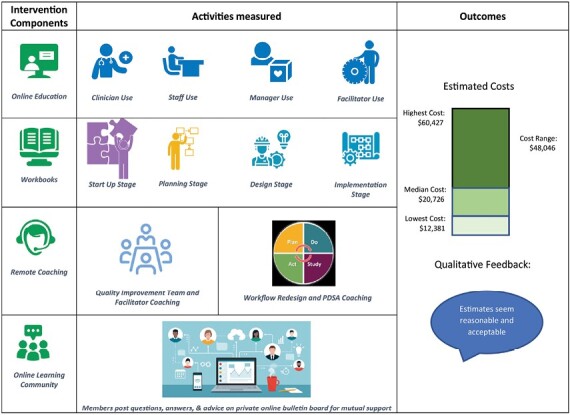
Study Sample: Eight primary care practice sites across the nation, purposefully selected to represent the study population

**Implications**

**Practice**: Our implementation cost estimates provide a practical starting point for the leadership of various healthcare organizations to project the costs of moving practices from co-located to more integrated behavioral health services.

**Policy**: Our study demonstrates the feasibility of financing the start-up of integration of behavioral health services into primary care and provides a foundation for crafting policies that facilitate the fiscal support of this type of integration.

**Research**: This pilot study can guide researchers to design and conduct more definitive experiments that measure the cost of implementing behavioral health integration across a broader set of outpatient environments.

## Background and Rationale

The integration of behavioral health in primary care practices ideally brings medical and behavioral health providers (BHPs) together, in one location and with a team-based approach that includes patients as key team members, with the capacity to contribute to the management of chronic conditions that drive morbidity, mortality, and health care costs [1, 2]. Behavioral health (BH) care includes a variety of services that have historically been isolated from each other and are brought together in a single model of care [3], including mental health treatment, substance use disorder care, health behavior change, and attention to family and other psychosocial factors that affect a person or a family unit. Integrated BH models approach care from a biopsychosocial approach, addressing stress, mood, lifestyle behaviors (e.g., diet, exercise, sleep, and substance misuse), as well as chronic pain, medication adherence, family dynamics, and broader psychosocial concerns.

Integrated behavioral health (IBH) is an approach to patient care that addresses both behavioral and medical needs within primary care settings [4]. It is well known that primary care has had difficulty responding to some complex patient needs such as behavioral and medical comorbidities [5]. Research supports an association between IBH and improved patient outcomes, but integration can be a challenge for primary care practices [2, 6]. A large, pragmatic, national study of an implementation strategy to improve integration, *Integrating Behavioral Health and Primary Care for Comorbid Behavioral and Medical Problems* (IBH-PC; https://clinicaltrials.gov/ct2/show/NCT02868983), aimed to test the effectiveness of a comprehensive practice-level intervention designed to increase the practice’s degree of IBH service, thereby improving outcomes in patients with multiple chronic conditions [7]. To complement these findings, the value-add of patient-centered outcomes is often evaluated through a fiscal lens, focusing mainly on the cost of the IBH-PC care model. To respond to the practical need in assessing the cost of implementing the intervention, as borne by the clinic sites, the study also assessed various aspects of clinic investments in time and cost associated with the implementation of the IBH-PC ­program, specifically the time invested by clinicians, leaders, and staff to participate in education, redesign, collaboration, and coaching activities calculated as salary-based costs. This comprehensive approach was intended to more fully appreciate the encompassing value proposition of IBH-PC.

## Brief Description of the IBH-PC Program

The IBH-PC program is a multi-faceted implementation strategy that supports complex change in a primary care setting to move practices from co-location of BH services toward greater integration, as measured by the “Practice Integration Profile,” a survey of at least four practice members’ perceptions of integration on such topics as workflow and clinical services [8–10]. Improvement of integration was supported by a toolkit-based implementation approach, the “Integration Toolkit.” The Integration Toolkit includes four components that are associated with successful organizational change and were provided at no cost to sites in the intervention arm:

1. An online educational curriculum of 4–14 hours per practice member, depending on clinic role, providing shared language, concepts, and skills specific to primary care practice roles (medical provider, behavioral health provider, clinical staff, practice manager, non-clinical staff, and internal quality improvement [QI] team facilitator);
2. Structured, team-based, practice redesign and implementation workbooks to guide a QI team and facilitator through a step-wise series of activities in distinct project stages (planning, redesign of workflows, and implementation of practice changes) and sub-stages;
3. Remote QI coaching services, providing clinic-specific support for a QI team and facilitator by experts in IBH, QI and workflow redesign; and
4. An online learning community, offering support from colleagues engaged in IBH improvement.

The Integration Toolkit is based on multiple components of the Expert Recommendations for Implementing Change (ERIC) project [11, 12], including education about integrated care, assessment for readiness to implement, technical assistance on implementation, team-based implementation, practice facilitation, patient and family engagement, innovation adaptation, small tests of change, measurement of results, scale up, and use of a learning collaborative. Portions of the Integration Toolkit were iteratively developed and refined in previous studies [13–16].

## Methods

From a group of 42 practices that participated in the entire IBH-PC study, we purposefully selected 8 intervention practices to provide a reasonably representative sample of the study population. We based our selection criteria on the number, type, and effort of medical providers, practice panel size, patient visit volume, number and effort of behavioral providers, National Committee for Quality Assurance (NCQA) Patient-Centered Medical Home (PCMH) status, proportion of adults on Medicare, type of practice (health center, health system-owned, clinician-owned, academic, etc.) and location of practice (urban, sub-urban, rural). We created four tiers of practices across these variables and assigned two practices to each tier, accordingly. We also identified replacement practices to address variations in their willingness to initiate participation or the possibility of attrition. In each of the 8 selected practices, we identified all employees who contributed to the IBH-PC integration process in any tangible manner. These became subjects of the econometric analyses. We conducted our pragmatic study from the perspective of clinic owners, in order to provide practical ballpark estimates of implementation costs in support of future decision-making.

We conducted an observational econometric cohort study that included 8 selected practice sites. Each practice identified a project champion as well as a QI team facilitator to support the redesign process during the active phase of the study. Following the active phase, we recruited these champions and facilitators to complete a Microsoft Excel-based Cost Assessment Tool (CAT) that we developed incrementally and systematically pilot-tested at the beginning of the project. Data reporters were not compensated for their participation and were free to withdraw from this part of the IBH-PC study with no obligation or penalty. They were allowed to work through each step of the CAT sequentially, at their own pace, entering data by specific practice roles (e.g., clinician, behavioral health provider, staff, office manager). As a result, the CAT captured close estimates of time-effort data related to implementing the step-wise IBH-PC program at those clinic sites. The CAT presented data fields to gather the amount of time spent (in minutes) with program activities outlined in the implementation workbook by each practice role and the number and type of practice participants performing these activities by project stage and sub-stage. The CAT tallied the time each practice member spent to complete all program steps across 4 project components, including IBH-PC program implementation tasks, remote QI coaching services, educational curricula, and learning community activities, over a 24-month period.

The CAT calculated sub-totals of time-effort and cost in U.S. dollars using median total compensation (MTC) rates appropriate for each practice’s ZIP code and the participants’ professional roles. MTC rates were obtained using the widely utilized and reliable salary.com online database by identifying specific job titles used consistently across the application that most closely matched each practice role in our project (e.g., Family Medicine Physician or Office Operations Senior Manager). We narrowed estimates to particular ZIP codes and cities where study practices were located and we included base salary, bonus, and the value of benefits to project each person’s total compensation. These were then used to calculate specific time-cost estimates according to each project-related task performed. The CAT also incorporated additional costs of supplies, equipment, capital, or other nonpersonnel costs, as well as time spent with education modules and remote QI coaching services associated with toolkit use. Time-effort data were then checked to ensure that they were complete, fell into allowable ranges, had face validity in terms of logical time estimates, and were appropriate to calculate econometric measures. Missing or erroneous information was corrected by working with QI team facilitators at each clinic site.

Primary and secondary econometric outcomes were estimated by calculating overall and subgroup means and medians for the 8 selected practices that encompassed total time, cost of individual program implementation steps, time and cost of the planning stage and the combined cost of redesign and implementation stages. To reflect subjects’ actual use of the Integration Toolkit which resulted in iterative use of the implementation stages, we combined the originally planned redesign and implementation stages into one unified stage. Cost estimates were adjusted for inflation during the 2-year data collection period using an inflation rate of 1.55% for calendar year 2018 and 2.49% for calendar year 2019 based on data published by the U.S. Bureau of Labor Statistics (https://www.bls.gov/cpi/data.htm). We have not applied any discounting in the process of adjusting our cost estimates.

We used Student’s *t*-test to compare means of continuous data and Fisher’s exact test to compare proportions of categorical data. Comparisons of the subsample of 8 practices to all other IBH-PC practices were conducted to show that the 8 practices were statistically representative of the larger study sample. Practice and patient population demographics were collected previously by the main IBH-PC study and were made available to our study team. Our study relied on retrospective staff estimates that went back approximately 1 year or less, focusing on the practice unit-level implementation of the Toolkit, since it was not feasible to conduct real-time observations (e.g., a time–motion study). Estimates did not include all associated costs, such as variable and opportunity costs. Implementation cost data were derived only from post-hoc surveys of practice staff actions taken and task-related investments committed by practices.

The perceived validity and face-value of estimates were checked via follow up conversations between data reporters and the larger study team. The study team made presentations of these outcomes to practice leadership and/or QI teams, asking for perceptions based on their knowledge of the clinic. The transcripts of informal feedback were reviewed by two study team members for consistency of interpretation.

## Results

The final set of 8 selected econometric study practices was located across the entire United States, including Hawai’i. Their patient panels ranged from 2,000 to 16,000; they saw between 4,200 and 32,000 patients per year; their NCQA PCMH status ranged from no certification to Level 3; they employed 3 to 13 primary care clinicians and 1 to 7 ­behavioral health providers. Four of the practices represented community health centers of various types, six practices included family medicine residencies (academic and nonacademic), and one practice was managed by a large private health system. Half of the practices were located in rural areas, two practices were urban and two served mainly suburban or peri-urban populations (see **Table 1**). One practice decided not to participate in the early phase of the study due to competing priorities and was replaced with an alternative practice that had closely matching characteristics.

**Table 1:** Comparison of Practice Characteristics by Study Sample

Practice Characteristics	Econometric study practices (*N* = 8) Mean (± SD) or proportion (%)	All other IBH-PC practices (*N* = 34) Mean (± SD) or proportion (%)	P Value^#^
Mean patient panel size	7,861 (±1451)	9,608 (±5344)	0.39
Mean patient encounters per year	19,644 (±10,958)	28,469 (±21,034)	0.26
Mean primary care provider count	8.3 (±3.0)	10.1 (±6.3)	0.43
Mean behavioral health provider count	2.6 (±2.3)	2.8 (±2.7)	0.85
Mean PIP* score at baseline	60.1 (±11.2)	58.9 (±15.1)	0.83
Proportion of adults on Medicare	23.0 (±18.7)	20.7 (±13.3)	0.69
Proportion of comm. health centers	4/8 (50.0%)	10/34 (29.4%)	0.29
Proportion of sites with residency	4/8 (50.0%)	11/34 (32.3%)	0.38
Proportion of academic sites	2/8 (25.0%)	3/34 (8.8%)	0.22
NCQA PCMH < level 2	4/8 (50.0%)	19/34 (55.8%)	0.70
Completion of IBH-PC study per protocol	8	34	1.00

^*^PIP: From the Practice Integration Profile survey that measures the level of behavioral health integration on a scale from 0-100.

^#^Means of continuous data were compared by Student’s t-test; Proportions of categorical data compared by Fisher’s exact test.

There were no statistically significant differences between the 8 selected econometric study practices and the rest of the practices (*N* = 34) within the complete IBH-PC study sample in geographical distribution (e.g., rurality), patient panel size and number of visits, clinician workforce (e.g., medical and behavioral health provider full time equivalent employee), specialty mix, adult Medicare ratio, level of baseline behavioral health integration, and completion of IBH-PC. The eight practices represented a moderately higher ratio of academic institutions (25% vs. 8.8%), training sites (50% vs. 32.3%), and community health centers (50% vs. 29.4%), but these differences were not statistically significant.

Similarly, no significant differences were observed between the econometric study patient subsample and the rest of the IBH-PC patient sample pertaining to mean age at baseline, ratio of 65-years old and older, and proportions of white race, male sex, Hispanic ethnicity, marital status, being in the workforce, low income, and college education (see **Table 2**).

**Table 2:** Comparison of Patient Characteristics by Study Sample

Patient characteristics	Econometric study practices (*N* = 8) Mean (± SD)	All other IBH-PC practices (*N* = 34) Mean (± SD)	P Value^*^
Mean age at baseline (years)	64.2 (±4.9)	65.7 (±4.4)	0.39
Age 65 and older at baseline (%)	47.7 (±16.8)	51.8 (±15.6)	0.51
Proportion of white race (%)	78.8 (±27.0)	76.9 (±19.0)	0.82
Proportion of male sex (%)	37.3 (±9.9)	36.5 (±6.8)	0.80
Proportion of Hispanic ethnicity (%)	11.3 (±8.2)	11.7 (±12.2)	0.92
Proportion of married state (%)	52.6 (±6.6)	46.6 (±14.5)	0.32
Proportion of working status (%)	30.9 (±9.9)	28.1 (±8.5)	0.41
Proportion of low income (%)	50.5 (±29.7)	48.3 (±20.8)	0.81
Proportion of college education (%)	44.8 (±18.8)	48.6 (±17.9)	0.60

^*^Means of continuous data were compared by Student’s t-test.

The IBH-PC Integration Workbooks outlined several project stages (i.e., Planning, Design, and Implementation) and the CAT was designed to collect econometric data accordingly, including all 4 project components conducted during these project stages. Since in several practices, IBH-PC program implementation followed a cyclical and iterative process, we have summed all costs incurred after the Planning Stage in each practice under an Implementation Stage cost estimate. The median cost of the Planning Stage was $10,258 across study sites (range: $4,625–$14,840), while the median cost of the Implementation Stage was $9,208 (range: $6,017–49,993).

The total median cost of implementing the entire IBH-PC program across all stages was $20,726 (range: $12,381–$60,427). Total cost estimates identified two practices in our sample with costs over twice of the median cost of the rest of the practices ($49,372 and $60,427 vs. $18,576, respectively). One of these was a large urban academic residency that chose to re-allocate 10% effort from a behavioral health provider to facilitate IBH improvement and the other was part of a large health system that opted for system-wide project implementation using centralized resources. A more detailed description of implementation costs are shown in **Table 3**.

**Table 3:** IBH-PC Program Implementation Cost Estimates in Eight Practices

Practice location	Practice type	Planning stage cost	Implement. stage cost	Total cost
Practice 1	Small Academic FM Residency (Rural)	$10,081	$9,687	*$19,768*
Practice 2	Small-Mid Community Health Ctr (Rural)	$14,840	$6,844	*$21,684*
Practice 3 ^*^	Large Academic FM Residency (Urban)	$13,433	$35,939	*$49,372*
Practice 4 ^#^	Mid-Size Health System Practice (Suburban)	$10,434	$49,993	*$60,427*
Practice 5	Mid-Large CHC FM Residency (Mid Rural Town)	$9,624	$14,390	*$24,014*
Practice 6	Mid-Size Academic FM Residency (Peri-Urban)	$11,367	$6,017	*$17,384*
Practice 7	Mid-Size Comm. Health Center (Small Rural Town)	$4,625	$7,756	*$12,381*
Practice 8	Mid-Size Community Health Center (Urban)	$7,529	$8,728	*$16,257*
Median Cost by Stage:		$10,258	$9,208	**$20,726**
Cost Range by Stage:		$10,215	$43,976	**$48,046**
	**Total Median Cost Without Outliers** **[3]** ** &** **[4]** **:**			***$18,576***
	**Total Cost Range Without Outliers** **[3]** ** &** **[4]** **:**			***$11,633***

^*^Large urban academic residency.

^#^One practice within a health system-wide implementation.

Leaders and QI team members from 39 of 42 practices (93%) participated in follow up presentations. Unstructured feedback suggested wide-spread agreement that the cost outcomes for their clinics were acceptable and reasonable and that the information would be helpful in making future plans to integrate care. According to one CEO, “Even the $60K (i.e*.,* the top of the range) doesn’t scare me, if I knew I was going to integrate my practice.”

## Discussion

Our study found that the total median cost of implementing the IBH-PC program, as outlined in the Integration Workbooks, ranged between $12,381 and $60,427. This included a Planning Stage (ranging from $4,625 to $14,840) and an Implementation Stage (ranging from $6,017 to 49,993) in 8 primary care practices. These costs were well within an expected range and they were deemed reasonable and acceptable by the participating organizations.

Recent studies continue to support the value of BH integration in primary care as measured by multiple patient and clinical care outcomes [17–27]. Furthermore, the effectiveness of behavioral treatment of chronic disease (the patient population under study in IBH-PC) has been supported by multiple studies [28–38]. Studies found successful implementation strategies for effective integration [39–42], of which toolkits were one example [43], although not yet supported by robust study designs reporting on patient outcomes [44].

Reporting on how the cost of implementing BH in primary care varies in methods and outcomes, Reiss-Brennan et al. [45] computed the cost of BH in Team-Based Care practices from internal payroll, accounting, and asset management data systems. One-time transition costs (infrastructure expansions, phones, computers) were examined separately from ongoing operational costs (labor expenses, care coordination costs, and quality incentives). These costs were described as “lower than the reduction in payments received by the ­delivery system,” meaning that reductions in utilization paid back the dollar cost of transition for team-based, integrated care. However, the authors also noted that implementation was resource-intensive in ways not measured in dollars (i.e., time invested by leadership, clinical and analytical providers and staff and information technology support). Our results supplement this work by capturing the investment time of all members of the practice in resource-intensive engagement. We agree with Reiss-Brennan et al. that a value-based approach to assessing such programs is needed to understand investments relative to outcomes over time and offer our method as one such approach.

Lang and Connell took a similar approach to evaluating the costs of evidence-based practice implementation which they too see as a critical and missing methodology for Dissemination and Implementation research [46]. They described their approach using a yearlong statewide dissemination of Trauma-Focused Cognitive Behavioral Therapy (TF-CBT) in child outpatient clinics at community mental health agencies in Connecticut through learning collaboratives. While the study was not focused on integrated care, this methodology recognized, as did ours, the differences between pre-implementation and implementation costs, to which they added sustainment (or operational) costs. They evaluated incremental implementation costs beyond what would be expected for “treatment as usual” by asking “(1) What are the incremental (additional) costs in dollars and staff time to a clinic implementing TF-CBT through a learning collaborative and (2) What implementation activities account for these costs?” The authors developed an “Implementation Cost Survey” (ICS) with 38 items to assess start-up incremental implementation costs of each clinic.

The ICS, similarly to the CAT, included learning sessions, when training was provided, and action periods, the time between learning sessions when the evidence-based practice was put into practice in a clinic. Like the CAT, staff time for each implementation activity was reported separately for clinicians, clinical supervisors, the site coordinator, and the senior leader to account for differences in responsibilities and salaries. Unlike the CAT, the ICS tracked implementation activities that included expenses beyond the practice site, such as, evidence-based practice specific supervision, data management, leadership/agency communication, travel, supplies, family partner (consumer participation on the implementation team), and non-billable time. The results of this 10-clinic study showed variability among clinics, with an average incremental implementation cost of $89,575, median $94,393, and range $34,697–$130,063. These costs, which are higher than those we found, are likely due to the inclusion of operational costs incurred during the maintenance phase of the project, as well as costs attributed to participants and activities outside the practice. This study emphasized the importance of studies on cost of implementation, as this is one of the most frequently cited barriers to dissemination and implementation despite being one of the least widely studied factors.

Closest to our approach of evaluating costs, Ritchie et al. used a structured, retrospective field log to identify the organizational cost of implementing primary care and mental health integration at 8 clinics in two VA medical center networks. However, this study focused only on the cost of external and internal facilitators, their support staff, and stakeholder participation time in facilitated activities over a 28 month period [47]. It did not include time spent by providers and staff on activities between facilitated events, time in education ­sessions, or engagement in online collaboration, as captured by the CAT. The organizational cost of salary support for facilitation activities, excluding travel salary support and expenses, was $236,263 in network “A” and $208,314 in network “C,” each of which had 4 clinics to support. The authors noted that complex implementations, such as integrated care, plus the implementation challenges in primary care settings, require substantial organizational investments which may vary by clinic.

Our study fills a gap in the sparse literature on costs of integrating behavioral health services into outpatient office settings. Reiss-Brennan calculated the cost of “team-based care” from internal financial systems which did not account for the substantial investment of leaders, providers, and staff to implement a new way of delivering care. Ritchie collected implementation time of facilitators and clinic stakeholders involved in facilitated events in support of integrated behavioral health in primary care through a field log completed by the facilitators. Lang developed a cost survey for the clinic administrator and implementation team, which captured the time spent in specific implementation activities to adopt a specific behavioral intervention, TF-CBT. The CAT is a field log that captures all time related to implementation, whether in facilitated events such as team meetings or other activities of leading and supporting change. This spans the phases of planning, design, and implementation of new workflows that reflect various organizational stages in conducting change.

Our findings are generally consistent with results of the two cost studies on implementation of complex change in ambulatory settings that reported these numbers. Our cost range of $12,381–$60,427 overlaps with Lang’s of $34,697–$130,063, which included maintenance costs and costs related to agents outside the practice and, accordingly, has a higher median ($20,726 for CAT *vs.* $94,393 Lang). Ritchie did not report costs at the clinic level but noted that each network included four clinics, leading to an average per clinic cost in network “A” of $59,066 and in network “B” of $52,079. The Integration Toolkit was engineered to support clinic-level and clinic-supported change with typically little system support (practices 3 and 4 in Table 3 being the exceptions), which supports its lower cost results. Higher integration costs in practice 3 and 4 can be explained by their greater size, different structure (e.g., urban academic residency), or deeper implementation (e.g., system-wide deployment approach) compared to the other 6 sites.

Our particular cost assessment approach was driven by several initial considerations. First, we established that the goal of this sub-study within the larger scope of the IBH-PC study was to provide a reasonable ballpark estimate of the cost associated with implementing *the specific program steps outlined* in the IBH-PC Integration Toolkit to help guide future executive decisions about this type of implementation. For this reason, our approach, by design, did not extend to including all costs (fixed, variable, operational, and opportunity costs) associated with moving a primary care practice from BH service co-location to greater integration. We believe this gives administrators and decision-makers an estimate of the investment needed to move the complex process of IBH a step forward in an ongoing progression of improvement. Second, we preferred a pragmatic measurement process which we deemed feasible and replicable by other investigators and healthcare organizations. These measurements were based on post-hoc surveys of actions taken and investments ­committed by practices, as opposed to *e.g.*, conducting tedious, costly and burdensome time-motion studies. We determined that the latter would not be feasible in the busy and stressed primary care practices involved in IBH-PC. Third, we focused our assessment on the local, practice unit-level implementation and did not extend our analyses to the wider health systems within which the implementation experiments were conducted. This secondary (“macro”) layer of assessment was beyond the scope of our study. Our results indicate that clinics considering a pragmatic approach to converting from on-site BH services to greater integration can expect to spend on the order of $20,000 about evenly split between planning and implementation efforts.

Primary care practices are complex adaptive systems that operate in a highly dynamic and multi-faceted environment [48]. Our study and a large body of research that has been done in this setting certainly support this theory. Even without factoring in major changes due to the coronavirus pandemic, our practice cohort experienced strong time trends and a significant number of major disruptive events acknowledged in the literature [49–51], including the loss and turnover of workforce, ownership changes, medical record system disruptions, major shifts in organizational strategies, and so on. This did not allow for a more prescriptive program implementation protocol and required environment-specific tailoring of the implementation and significant flexibility to adapt to emerging developments in each practice environment. For this reason, for example, 3 of the 8 participating practices condensed Stages 2 and 3 of the implementation plan after a relatively uniform planning stage (Stage 1), conducting a series of Plan-Do-Study-Act cycles to implement the elements of the IBH-PC program and iterating the steps outlined in the last 2 stages. As another example, one of the large health system practices employed a QI facilitator who was an employee of the health system and co-facilitated the IBH-PC program across all of its sites in the active arm, including the practice which was part of our study. This likely resulted in a somewhat different economy of scale and implementation dynamics in this particular practice. Due to these factors, we combined the originally planned two implementation stages (Stages 2 and 3) into one stage for the cost assessment and presented them as the second stage of implementation.

## Limitations

Our study has several notable limitations. First, our exploration of the time and cost of this intervention was limited to 8 of the 42 clinics participating in the IBH-PC study. While the small sample size does not provide an exhaustive picture of the costs that every clinic incurred, the clinics that participated in the cost assessment were representative of the full IBH-PC sample regarding practice characteristics and patient population (see Table 2).

Second, this study was not powered to infer any relationship between cost and program effectiveness. This study seeks to understand the cost of implementing the toolkit and does not assess whether a more resource intensive implementation is correlated with increased integration. Exploring the relationship between the intensity of the intervention and its relationship to effectiveness and cost is an opportunity for future research. We also did not perform any sensitivity or uncertainty analyses which were beyond the scope of our study. This implies that there are three key sources of uncertainties in our approximations that include: 1) the precision of time estimates recalled for each task performed; 2) the actual total compensation each practice member received for their work, and; 3) potential unidentified or intangible costs linked to the introduction of new work processes. We believe, however, that the estimates we have developed are sufficient and acceptable for the limited purpose of guiding practice owners as they make a decision on whether to invest in behavioral health services in their organizations.

Third, to reduce burden to sites, data were collected retrospectively via self-report at the end of the two-year intervention and observation period. This resulted in about a median of 2.5 (range: 0–8) months lag time between the close of the observation period when tasks were performed and documenting their effort-estimates. While this approach was more acceptable to practices, as it required less time and documentation, retrospective data collection may be less accurate than prospective observations and limits extensive follow up data collection, such as in-depth interviews. However, our team made a concerted effort to obtain high-quality data and confirm the face validity of estimates with practice sites and the study team.

Fourth, data were collected by one person, the practice team facilitator or primary champion, at the clinic. While not engaging every team member in the reporting process does pose the risk of missing data, the study team deemed that risk to be low. The data reporter was certainly central to the project, giving them the best overall picture of the team and their work. They were also encouraged to confer with team members if they lacked clarity on time spent on any activities.

Fifth, cost estimates only included actual costs to clinics, not costs incurred by the study team. The intervention included remote QI coaching services, which were not accounted for in this study, as coaching was provided at no charge to practices as a part of the intervention. Practices that desire coaching support may incur additional expenses beyond those calculated in this study depending on the services provided by their states’ Area Health Education Centers or similar support services. States vary widely in their support of primary care practice change and it is beyond the scope of this study to evaluate alternatives that are determined by state policy. As another example, some expenses were determined by health system characteristics and expectations (e.g., amount and use of training resources) which will again vary independently from the use of the Integration Toolkit. It is also important to consider cost savings as a means to offset implementation costs. While our study did not capture this, a previous review by Muse, et al. (2017) demonstrated that IBH resulted in shorter PCP visits as providers were able to hand-off patients to the BH provider [52].

Sixth, the study team focused only on direct expenses incurred by practices and did not collect data on any changes in billing revenue sustained during the intervention, which may have added to or lessened the net cost to practices. All participating practices had a BH provider prior to participating in the IBH-PC study. This study did not account for the potentially significant cost of recruiting and onboarding BH providers for practices without BH providers to undertake the IBH-PC program.

Finally, legal restrictions in place at the time of the funding award prohibited the development of formal cost-effectiveness analysis. However, a detailed analysis of program effectiveness is in preparation in the context of the larger IBH-PC study.

## Conclusions and Future Directions

We believe that despite some limitations, our study provides a reasonable and actionable ballpark estimate of the most direct costs of implementing a behavioral health integration program using a structured, team-based approach along the lines of IBH-PC in primary care settings. In particular, incremental costs that practices may incur to secure the participation of clinicians and staff and commit personnel time to a behavioral health integration effort are well represented in our study. Since the lion’s share of program implementation cost is most frequently personnel time, it is encouraging that the total median cost of the IBH-PC behavioral health integration effort was around $21,000, ranging between $12,000 and $60,000 and that these results were found to be acceptable and helpful to almost all participating sites in later presentations.

The original focus of the IBH-PC study was to track outcomes of a toolkit-based implementation (https://sites.google.com/view/ibhpc/home) given the growing trend of integrated behavioral health. Midway through the study, the COVID-19 pandemic resulted in significant changes in clinic operations in many of the practices. As a result, our secondary study on implementation costs has become even more relevant as the need for behavioral health services has sky-rocketed during COVID-19 [53] and this trend will likely have a long tail despite decreasing case counts.

Moving forward, the implementation cost data can be used to advocate for IBH-PC programs in a number of ways. As mentioned above, a $60K start-up cost does not seem unreasonable for CEOs and demonstrates that IBH has an inherent value proposition in which its benefits are acknowledged to outweigh its implementation costs. In the past, it has been difficult to create a business plan for IBH-PC because so many of the costs tended to be hidden. The cost breakdown generated in our study provides a framework for BH providers and financial managers to create more informed BH integration proposals. Additionally, the availability of federal funding related to the current pandemic could also be used to support new healthcare improvement initiatives. A price-point for implementation costs could be used to secure federal funding for start-up costs incurred by primary care practices without integrated behavioral health services, particularly at this time when there is an increased demand for mental and social health services.
